# FoxP3^+^ CD8 T-cells in acute HIV infection and following early antiretroviral therapy initiation

**DOI:** 10.3389/fimmu.2022.962912

**Published:** 2022-07-29

**Authors:** Alexis Yero, Tao Shi, Jean-Pierre Routy, Cécile Tremblay, Madeleine Durand, Cecilia T. Costiniuk, Mohammad-Ali Jenabian

**Affiliations:** ^1^ Department of Biological Sciences and CERMO-FC Research Centre, Université du Québec à Montréal (UQAM), Montreal, QC, Canada; ^2^ Research Institute of McGill University Health Centre, Montreal, QC, Canada; ^3^ Chronic Viral Illness Service, Department of Medicine, Glen Site, McGill University Health Centre, Montreal, QC, Canada; ^4^ Centre hospitalier de l'Université de Montréal (CHUM) Research Centre, Montreal, QC, Canada; ^5^ Department of Microbiology, Infectiology and Immunology, Faculty of Medicine, Université de Montréal, Montreal, QC, Canada

**Keywords:** CD8 regulatory T cells (CD8 Tregs), acute HIV infection, early antiretroviral therapy (ART), FoxP3, TGF-β1, CD39

## Abstract

**Objectives:**

Besides CD4 regulatory T-cells (Tregs), immunosuppressor FoxP3^+^ CD8 T-cells are emerging as an important subset of Tregs, which contribute to immune dysfunction and disease progression in HIV infection. However, FoxP3^+^ CD8 T-cell dynamics in acute HIV infection and following early antiretroviral therapy (ART) initiation remain understudied.

**Methods:**

Subsets of FoxP3^+^ CD8 T-cells were characterized both prospectively and cross-sectionally in PBMCs from untreated acute (n=26) and chronic (n=10) HIV-infected individuals, early ART-treated in acute infection (n=10, median of ART initiation: 5.5 months post-infection), ART-treated in chronic infection (n=10), elite controllers (n=18), and HIV-uninfected controls (n=21).

**Results:**

Acute and chronic infection were associated with increased total, effector memory, and terminally differentiated FoxP3^+^ CD8 T-cells, while early ART normalized only the frequencies of total FoxP3^+^ CD8 T-cells. We observed an increase in FoxP3^+^ CD8 T-cell immune activation (HLADR^+^/CD38^+^), senescence (CD57^+^/CD28^-^), and PD-1 expression during acute and chronic infection, which were not normalized by early ART. FoxP3^+^ CD8 T-cells in untreated participants expressed higher levels of immunosuppressive LAP(TGF-β1) and CD39 than uninfected controls, whereas early ART did not affect their expression. The expression of gut-homing markers CCR9 and Integrin-β7 by total FoxP3^+^ CD8 T-cells and CD39^+^ and LAP(TGF-β1)^+^ FoxP3^+^ CD8 T-cells increased in untreated individuals and remained higher than in uninfected controls despite early ART. Elite controllers share most of the FoxP3^+^ CD8 T-cell characteristics in uninfected individuals.

**Conclusions:**

Although early ART normalized total FoxP3^+^ CD8 T-cells frequencies, it did not affect the persistent elevation of the gut-homing potential of CD39^+^ and LAP(TGF-β1)^+^ FoxP3^+^ CD8 T-cell, which may contribute to immune dysfunction.

## Introduction

Immunosuppressive CD8 T-cells are a heterogeneous group of suppressor T-cells with various origins, phenotypic characteristics, and suppressive mechanisms. Despite 50 years since their discovery (1), our understanding of the regulation and functions of these cells remains limited compared to their CD4 Treg counterparts, mainly due to the lack of specific characterization markers. However, various studies have revealed the undeniable role of immunosuppressive CD8 T-cells in cancer, autoimmune diseases, transplantation, and infectious diseases (2–6). Indeed, several CD8 T-cell populations with immunosuppressive capacity have been described, including those expressing FoxP3, the master transcription factor of Tregs, or other populations with immunosuppressive features in the absence of FoxP3 expression (7–9).

In physiological conditions, human CD4 T-cells usually express FoxP3 at higher levels than CD8 T-cells, and the frequencies of CD4^+^FoxP3^+^ T-cells are more elevated compared to FoxP3-expressing CD8 T-cells (around a 50-fold difference) (3, 10, 11). However, FoxP3 expression is crucial for the stability and functions of FoxP3^+^ CD8 T-cells (12, 13). Lim et al. demonstrated the presence and increased frequencies of FoxP3^+^ CD8 T-cells in the blood of HIV-infected individuals compared to uninfected controls (14, 15). They found a link between HIV disease progression and immune activation with the proportions of CD8^+^FoxP3^+^ T-cells while showing that FoxP3-expressing CD4 and CD8 T-cells in HIV-infected people are phenotypically distinct (15). SIV/HIV infections are associated with an increase in the frequencies of FoxP3^+^ CD8 T-cells that positively correlate with plasma viral load (VL), which negatively impact antiviral immune responses and contribute to HIV disease progression by inhibition of effector T-cell proliferation and cytokines secretion (15–18). Moreover, FoxP3^+^ CD8 T-cells induced after vaccination were critical in controlling SIV infection in Rhesus macaques (RM) by reducing CD4 T-cell activation and viral replication (19, 20). In a single report, higher FoxP3^+^ CD8 T-cell frequencies and absolute numbers were also observed in the blood of elite controller (EC) SIV-infected Indian RMs (18). However, the dynamics of FoxP3^+^ CD8 T-cells during acute HIV infection remain understudied.

Several subsets of FoxP3^+^ CD8 T-cells expressing highly immunosuppressive markers such as cytotoxic T lymphocyte antigen 4 (CTLA-4), programmed cell death protein-1 (PD-1), CD39, and transforming growth factor-beta 1 (TGF-β1) have been described (16, 17, 21–23). FoxP3^+^ CD8 T-cells express high levels of CTLA-4, which is needed for viral suppression in SIV-infected RMs (16, 17). Besides, CTLA-4 is required for FoxP3^+^ CD8 T-cells expansion, activation, and maintenance since the interaction CTLA-4/B7 promotes indoleamine 2,3-dioxygenase (IDO) expression by dendritic cells, further favoring the generation of CTLA-4^+^ FoxP3^+^ CD8 T-cells (22). PD-1 expression increases during SIV/HIV infections in correlation with immune activation, VL, and low CD4 T-cell count (24). PD-1/PD-1L contributes to the immunosuppressive functions of FoxP3^+^ CD8 T-cells (21). The ectonucleotidase CD39 hydrolyzes inflammatory ATP into ADP and AMP, followed by the generation of immunosuppressive adenosine in an orchestra with CD73 (25–27). The expression of CD39 by FoxP3^+^ CD8 T-cells is crucial for viral suppression in SIV-infected RMs (16). Fenoglio et al. found a positive correlation between the levels of CD39-expressing CD8 T-cells and VL, CD4 T-cell count and immune activation, suggesting their link with HIV disease progression (28). Furthermore, increased FoxP3^+^ CD8 T-cells in SIV infection correlated positively with TGF-β1 production (23). TGF-β1 limits effector T-cell proliferation while promoting the differentiation of both CD4 and FoxP3^+^ CD8 T-cells (12, 29, 30). TGF-β1 is first generated as a pro-TGF-β1, which is then cleaved to form a dimeric pro-peptide known as a latency-associated peptide (LAP), which binds non-covalently with mature TGF-β1 to prevent TGF-β1 binding to its receptor and subsequent activation (31). TGF-β1 production stimulates collagen-1 deposition and progressive lymphoid tissue fibrosis in SIV/HIV infections, starting during the acute infection (32, 33). Notably, TGF-β1-expressing CD8 T-cells are major contributors to fibrosis of lymph nodes and gut mucosal tissues during HIV infection regardless of the stage of the disease, antiretroviral therapy (ART) or disease outcome (34, 35).

Early ART initiation upon HIV exposure is highly recommended in clinical practice since it improves CD4 T-cell recovery and reduces VL and immune activation (36, 37). One study showed decreased FoxP3^+^ CD8 T-cell frequencies following early short-term ART in SIV-controllers RMs (38). Our team has recently reported that early ART initiation at four days post-infection can normalize CD39^+^ FoxP3^+^ CD8 T-cell frequencies in blood and mesenteric lymph nodes of progressor SIV-infected RMs (39). Moreover, we also recently reported an increase in total CD4 Tregs frequencies, which was normalized by early ART, while the frequencies of immunosuppressive CD4 Tregs-expressing CD39 and LAP(TGF-β1) with potential migration to the gut remained higher despite ART (40). However, to date, FoxP3^+^ CD8 T-cell dynamics during acute HIV infection and the impact of early ART initiation remain understudied.

Herein, we prospectively and cross-sectionally evaluated the dynamic of FoxP3^+^ CD8 T-cells during HIV infection and following early ART initiation in the acute phase. We found that despite decreasing frequencies of total FoxP3^+^ CD8 T-cells, early ART initiation failed to decrease the expansion of FoxP3^+^ CD8 T-cells with highly immunosuppressive functions and their potential migration to the gut, which may contribute to immune dysfunction and disease progression.

## Material and methods

### Study population

Frozen peripheral blood mononuclear cells (PBMCs) from HIV-infected individuals and uninfected controls were obtained from Montreal Primary and Slow Progressors HIV Infection cohorts and McGill University Health Centre. A total of 105 individuals were included in our study and our study has been carried out in both cross-sectional and longitudinal manners. In the cross-sectional analysis, 26 study participants had acute HIV infection, which was defined as being within 180 days after the estimated date of HIV infection (median (IQR) 90 (43–126) days). Individuals with chronic infection who had been infected for more than a year were left untreated (n=10) or given ART (n=11). HIV ECs (n=18) with CD4 count higher than 500 cells/ml in the absence of any treatment and undetectable plasma VL for at least 7 years, and 20 HIV-uninfected controls were also included in the cross-sectional analysis (**Table 1**). In addition, we followed longitudinally 20 acutely infected individuals overtime, ten of whom had started ART during the acute infection (median (IQR) 165 (97–212) days), and the other ten were left untreated (**Table 1**). Of note, our cross-sectional analysis did not include follow-up specimens from the longitudinal cohorts.

**Table 1:** Clinical characteristics of study groups.

	Cross-sectional participants						Longitudinal participants			
							Untreated		ART-Treated	
Characteristics	Non-infected (n=20)	Acute (n=26)	Chronic ART-(n=10)	Chronic ART+ (n=11)	EC (n=18)	*p-values*	Acute (n=10)	Chronic ART- (n=10)	Acute (n=10)	Chronic ART+ (n=10)
**Age, years** **[median (IQR)]**	39^d^ (30.75-47)	39.5^f,g^ (32.75-43)	32.5^h,i^ (26-39.75)	51^f,h^ (41–60)	49^d,g,i^ (32-55.5)	0.0006	39.5 (35.50-43.25)	39.5 (37.75-43)	36 (29.75-46.50)	36.5 (29-46.5)
**Male sex, n (%)**	15^a^ (75%)	26^a,g^ (100%)	10^i^ (100%)	11^j^ (100%)	10^g,i,j^ (55.6%)	0.0002	10 (100%)	10 (100%)	10 (100%)	10 (100%)
**CD4^+^ T-cells count, cells/µl** **[median (IQR)]**	632^b^ (463.5-775)	460^g^ (380–610)	440^b,i^ (255–543)	603 (400–847)	730^g,i^ (638.5-900)	0.001	515 (419-767.5)	595 (287.5-813.8)	450^l^ (272.5-561.3)	521^l^ (377.5-795)
**CD8^+^ T-cells count, cells/µl** **[median (IQR)]**	197^a,b,c,d^ (153-428.5)	996^a^ (640–1630)	750^b^ (629-1133)	743^c^ (433.3-1192)	739^d^ (604-1040)	0.0002	830^k^ (615-1170)	953^k^ (705-1915)	1019 (580-1708)	655 (531-1081)
**CD4/CD8 ratio** **[median (IQR)]**	2.82^a,b,c,d^ (1.41-4.19)	0.46^a,f,g^ (0.21-1.14)	0.48^b,h,i^ (0.40-0.61)	0.87^c,f,h^ (0.60-1.81)	0.95^d,g,i^ (0.80-1.43)	< 0.0001	0.56^k^ (0.40-1.35)	0.50^k^ (0.32-0.85)	0.40^l^ (0.19-0.81)	0.69^l^ (0.40-1.24)
**Nadir CD4^+^ T-cells count, cells/µl** **[median (IQR)]**	NA	330 (257.8-500)	310 (245-423.5)	334 (297.8-533.5)	551.5 (301.5-624.8)	0.30	365 (297.5-525)	NA	258.5 (207.5-530)	NA
**Viral load, log_10_ copies/mL** **[median (IQR)]**	NA	4.36^f,g^ (3.82-5.50)	4.56^h,i^ (3.74-3.98)	1.60^f,h^ (1.60-1.60)	1.65^g,i^ (1.60-1.69)	< 0.0001	4.07^k^ (3.54-4.37)	4.60^k^ (4.01-5.20)	4.40^l^ (3.92-5.77)	1.70^l^ (1.67-1.70)
**Duration of infection, years** **[median (IQR)]**	NA	0.25^e,f,g^ (0.12-0.35)	2.55^e,h,i^ (1.54-4.26)	12.40^f,h^ (4.99-19.33)	15.3^g,i^ (7.87-21)	< 0.0001	0.22^k^ (0.11-0.36)	2.19^k^ (2.12-2.39)	0.28^l^ (0.13-0.39)	2.27^l^ (2.00-2.65)
**Time of ART initiation years post-infection [median (IQR)]**	NA	NA	NA	1.11 (0.49-2.02)	NA		NA	NA	NA	0.46 (0.27-0.59)
**Duration of ART, years** **[median (IQR)]**	NA	NA	NA	14.58 (3.56-20.73)	NA		NA	NA	NA	1.72 (1.43-199)

Results are shown as median and interquartile range (IQR).

NA, not applicable; EC, Elite controllers.

p-values come from comparing the six groups using the Kruskal-Wallis test. Significant differences (p < 0.05) following Mann–Whitney U test or Fisher’s test are mentioned as follow:

a : Non-infected vs Acute,

b : Non-infected vs Chronic (ART-),

c : Non infected vs Chronic (ART+),

d : Non-infected vs EC

e : Acute vs Chronic (ART-),

f : Acute vs Chronic (ART+),

g : Acute vs EC,

h : Chronic (ART-) vs Chronic (ART+),

i : Chronic (ART-) vs EC,

j : Chronic (ART+) vs EC, Significant differences (p < 0.05) following Wilcoxon signed-rank test are mentioned as follow:

k : Acute vs Chronic (ART-),

l : Acute vs Chronic (ART+)

### Ethical considerations

The Ethical Review Board of the Université du Québec à Montréal (UQAM) gave their approval to this study (#2014-452), which followed the Declaration of Helsinki. All study participants signed a written informed consent form before blood collection.

### Flow cytometry analysis

Multiparameter flow cytometry was performed on thawed PBMCs. For immunological staining, the optimal concentrations of fluorochrome-conjugated antibodies were used in 3 independent panels of 14 colors each, as shown in **Supplementary Table 1**. The LIVE/DEAD Fixable Aqua Dead Cell Stain Kit (Invitrogen, Oregon, USA) was used to eliminate dead cells from the analysis. After extracellular staining, cells were permeabilized with the Transcription Factor Buffer Set (BD Bioscience, New Jersey, USA) and further stained intracellularly for FoxP3 and CTLA-4. The data was collected using a three-laser BD LSR Fortessa X-20 cytometer, and the results were analyzed using FlowJo V10.8.1 (Oregon, USA).

### Statistical analysis

GraphPad Prism V6.01 (California, USA) was used for statistical analysis. The results are shown as medians with an interquartile range (IQR) throughout the text. The distribution of variables was initially determined by the Kolmogorov–Smirnov test. The Kruskal–Wallis test was then used to evaluate any statistically significant differences between the five study groups. Nonparametric Mann-Whitney was used for unpaired variables, while the Wilcoxon rank tests were used for paired analysis. The correlation between variables was determined using the Spearman correlation coefficient test. Only statistical significances (p<0.05) are presented in the figures (*, P < 0.05; **, P < 0.01; ***, P < 0.001; ****, P < 0.0001).

## Results

### Characteristics of the study populations

ECs and chronically infected participants on ART were older than individuals in other study groups and had been infected for a longer period of time (**Table 1**). In chronic infection, ART restored CD4 T-cell count but was unable to normalize both CD8 T-cell levels and CD4/CD8 ratio compared to the uninfected group. ECs had similar CD4 T-cell count to those of uninfected individuals but had higher CD8 T-cell count and lower CD4/CD8 ratio. Chronically infected individuals on ART in the cross-sectional study were significantly older (median age: 51 *versus* 36.5 years, Mann-Whitney p= 0.04), had a longer duration of HIV infection (median: 12.7 *versus* 2.27 years, p< 0.0001), and had been longer on ART (median: 14.58 *versus* 1.72 years, Mann-Whitney p= 0.0002) than ART-treated chronically infected individuals in the longitudinal group. Furthermore, ART was initiated earlier in the longitudinal group (median years of ART initiation post-infection: 1.11 *versus* 0.46 years, Mann-Whitney p= 0.05). We thus evaluated the effect of early ART initiation on FoxP3^+^ CD8 T-cell in the longitudinal analysis. Early ART initiation improved CD4 T-cell count (Wilcoxon p= 0.04) and CD4/CD8 ratio (Wilcoxon p=0.002). Moreover, there was no significant difference in clinical characteristics between two (n=10) acute individuals in untreated and ART-treated longitudinal specimens.

### HIV infection is associated with a rapid expansion of total FoxP3^+^ CD8 T-cells, which was normalized by early ART initiation

**Figure 1:**
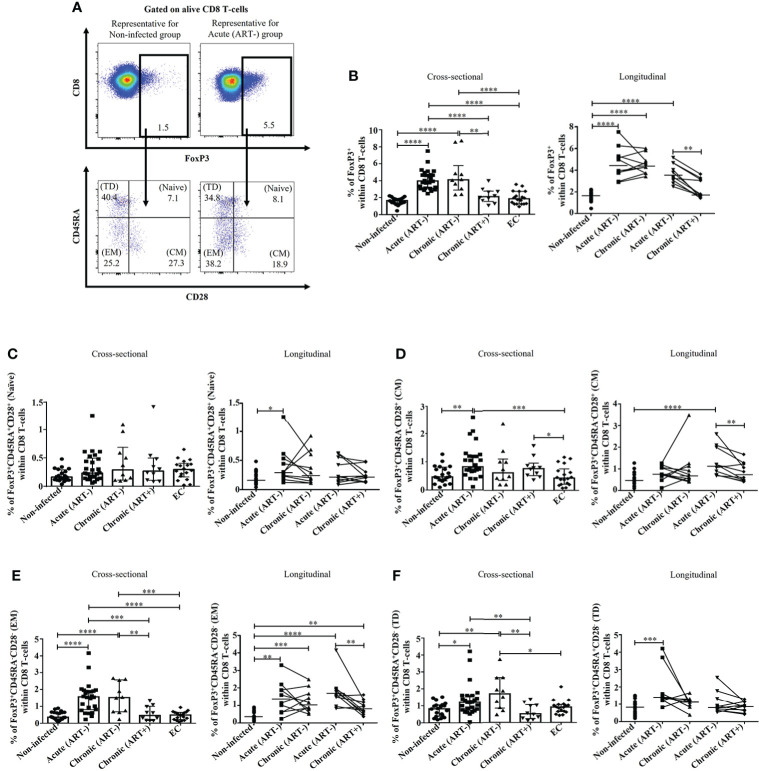
Effect of early ART initiation on total FoxP3^+^ CD8 T-cells frequencies and memory subsets. **(A)** Gating strategies used in flow cytometry to define total FoxP3**^+^** CD8 T-cells and FoxP3**^+^** CD8 T-cells memory subsets within CD8 T-cells. **(B)** Percentages of total FoxP3**^+^** CD8 T-cells. Frequencies of **(C)** naïve (CD45RA^+^CD28^+^), **(D)** central memory (CM, CD45RA^-^CD28^+^) **(E)** effector memory (EM, CD45RA^-^CD28^-^), and **(F)** terminally differentiated (TD, CD45RA^+^CD28^-^) FoxP3**^+^** CD8 T-cells subsets. Statistical significance is indicated in the figures as follow: *, P < 0.05; **, P < 0.01; ***, P < 0.001; ****, P < 0.0001. Differences among five study groups was determined by nonparametric Mann-Whitney rank test for unpaired variables, while the Wilcoxon rank tests were used for paired variables in the longitudinal study. Sample size in cross-sectional analysis: non-infected n=20, Acute n=26, Chronic ART- n=10, Chronic ART+ n=11, EC n=18. Sample size in longitudinal analysis: non-infected n=20, ART- n=10, ART+ n=10.

Untreated HIV infection increased FoxP3^+^CD8^+^ T-cells frequencies beginning in the acute phase compared to uninfected individuals (p< 0.0001 in both cross-sectional and longitudinal analysis), which was normalized by early ART initiation (**Figures 1A, B**). The frequency of total FoxP3^+^ CD8 T-cells was inversely correlated with CD4 T-cell count and CD4/CD8 ratio and positively with plasma viral load and both CD4 and CD8 immune activation (**Table 2**). Total FoxP3^+^ CD8 T-cells in ECs were significantly lower than untreated HIV-infected individuals (p< 0.0001 for both acute and ART- chronic) like uninfected controls (**Figures 1A, B**).

**Table 2:** Correlation between clinical and immunological parameters and CD4 T-cell count, CD4/CD8 ratio, CD4 activation (CD4^+^HLA-DR^+^CD38^+^), CD8 activation (CD8^+^HLA-DR^+^CD38^+^), plasma viral load (log_10_/ml), and duration of ART (years).

	CD4 T-cell count (cells/µl)		CD4/CD8 ratio		CD4 activation (HLA-DR^+^CD38^+^)		CD8 activation (HLA-DR^+^CD38^+^)		Plasma viral load (log_10_/ml)		Duration of treatment (years)	
	r	p	r	p	r	p	r	p	r	p	r	p
**CD8^+^FoxP3^+^**	**-0.424**	**0.0001**	**-0.4236**	**0.0001**	**0.6332**	**< 0.0001**	**0.678**	**< 0.0001**	**0.6005**	**< 0.0001**	-0.1089	0.68
**CD8^+^FoxP3^+^CD45RA^+^CD28^+^ (Naïve)**	0.07659	0.51	0.1419	0.22	0.1853	0.09	0.1797	0.1	-0.06796	0.59	-0.3841	0.14
**CD8^+^FoxP3^+^CD45RA^-^CD28^+^** **(CM)**	-0.1267	0.27	-0.06221	0.59	**0.2651**	**0.01**	**0.4002**	**0.0002**	0.2394	0.06	-0.2987	0.25
**CD8^+^FoxP3^+^CD45RA^-^CD28^-^** **(EM)**	**-0.4184**	**0.0002**	**-0.5109**	**< 0.0001**	**0.4998**	**< 0.0001**	**0.5**	**< 0.0001**	**0.597**	**< 0.0001**	0.03091	0.91
**CD8^+^FoxP3^+^CD45RA^+^CD28^-^** **(TD)**	-0.09472	0.41	-0.1506	0.19	**0.3426**	**0.001**	0.171	0.11	0.233	0.06	-0.2471	0.35
**CD8^+^FoxP3^+^CD38^+^HLA-DR^+^**	**-0.4041**	**0.0003**	**-0.514**	**< 0.0001**	**0.7546**	**< 0.0001**	**0.7502**	**< 0.0001**	**0.6481**	**< 0.0001**	-0.4812	0.05
**CD8^+^FoxP3^+^CD57^+^CD28^-^ (Senescent)**	**-0.3267**	**0.004**	**-0.5553**	**< 0.001**	**0.5502**	**< 0.0001**	**0.4332**	**< 0.0001**	**0.452**	**0.0002**	-0.5	0.05
**CD8^+^FoxP3^+^PD-1^+^**	**-0.4425**	**< 0.0001**	**-0.4824**	**< 0.0001**	**0.6112**	**< 0.0001**	**0.6657**	**< 0.0001**	**0.6954**	**< 0.0001**	-0.06034	0.81
**CD8^+^FoxP3^+^CTLA-4^+^**	-0.1377	0.23	**-0.2272**	**0.04**	**0.3825**	**0.003**	**0.5703**	**< 0.0001**	0.08438	0.51	0.2471	0.35
**CD8^+^FoxP3^+^CD39^+^**	-0.1306	0.26	-0.11	0.34	**0.4296**	**< 0.0001**	**0.5198**	**< 0.0001**	0.0468	0.71	**-0.5353**	**0.03**
**CD8^+^FoxP3^+^LAP(TGF-β1)^+^**	-0.125	0.28	-0.2067	0.07	**0.287**	**0.008**	**0.4102**	**0.0001**	-0.1189	0.35	0.3353	0.2
**CD8^+^FoxP3^+^CD39^+^LAP(TGF-β1)^+^**	-0.01223	0.91	0.03104	0.79	**0.2494**	**0.02**	**0.3246**	**0.002**	-0.1501	0.24	-0.25	0.34
**CD8^+^FoxP3^+^CCR4^+^**	-0.2196	0.05	-0.1122	0.33	**0.2996**	**0.005**	**0.4384**	**< 0.0001**	0.119	0.3569	0.02504	0.92
**CD8^+^FoxP3^+^CCR5^+^**	0.000274	0.99	0.02539	0.82	0.2145	0.05	**0.3031**	**0.005**	**0.2602**	**0.04**	-0.1441	0.59
**CD8^+^FoxP3^+^CCR6^+^**	**-0.3175**	**0.005**	**-0.2807**	**0.01**	**0.5175**	**< 0.0001**	**0.4891**	**< 0.0001**	**0.4594**	**0.0002**	0.07959	0.76
**CD8^+^FoxP3^+^CXCR3^+^**	**-0.3366**	**0.003**	**-0.4452**	**<0.0001**	**0.6352**	**< 0.0001**	**0.7059**	**< 0.0001**	**0.54**	**< 0.0001**	-0.02647	0.92
**CD8^+^FoxP3^+^CCR9^+^**	**-0.2133**	**0.06**	**-0.2591**	**0.02**	**0.5683**	**< 0.0001**	**0.6669**	**< 0.0001**	**0.3099**	**0.01**	**-0.7235**	**0.002**
**CD8^+^FoxP3^+^Integrin β7^+^**	**-0.3791**	**0.0007**	**-0.4641**	**< 0.0001**	**0.5138**	**< 0.0001**	**0.6631**	**< 0.0001**	**0.4482**	**0.0003**	-0.2931	0.26
**CD8^+^FoxP3^+^CCR9^+^CD39^+^**	-0.1289	0.26	-0.1451	0.21	**0.4407**	**< 0.0001**	**0.5628**	**< 0.0001**	0.139	0.28	**-0.5284**	**0.03**
**CD8^+^FoxP3^+^CCR9^+^LAP(TGF-β1)^+^**	-0.1275	0.27	-0.1418	0.22	**0.4638**	**< 0.0001**	**0.5614**	**< 0.0001**	0.1825	0.15	**-0.571**	**0.02**
**CD8^+^FoxP3^+^Integrin β7^+^CD39^+^**	-0.1667	0.15	-0.1665	0.15	**0.3779**	**0.0004**	**0.5338**	**< 0.0001**	**0.2866**	**0.02**	**-0.546**	**0.03**
**CD8^+^FoxP3^+^Integrin β7^+^LAP(TGF-β1)^+^**	-0.1987	0.08	**-0.2806**	**0.01**	**0.3916**	**0.0002**	**0.5249**	**< 0.0001**	0.1612	0.21	0.3265	0.21
**CD28^-^PD-1^+^ CD8 T-cells**	-0.1828	0.11	**-0.2973**	**0.009**	**0.2920**	**0.007**	**0.4417**	**<0.0001**	-0.0969	0.45	-0.09706	0.72
**CD28^-^CD39^+^ CD8 T-cells**	-0.03237	0.78	-0.2038	0.07	**0.2188**	**0.04**	**0.2443**	**0.02**	-0.0443	0.73	**-0.5107**	**0.04**

p-values come from the comparison of clinical and immunological parameters with CD4/CD8 ratio, CD4 activation (CD4^+^HLA-DR^+^CD38^+^), CD8 activation (CD8^+^HLA-DR^+^CD38^+^), plasma viral load (log10/ml), and duration of ART (years) by using the Spearman correlation coefficient test.

Significant differences (p < 0.05) are highlighted in Bold.

We observed a marked heterogeneity in FoxP3^+^ CD8 T-cell subsets based on CD28 and CD45RA expression (27, 41) in untreated HIV-infected individuals compared to ART-treated and uninfected controls (**Figure 1**). The frequencies of naïve (CD45RA^+^CD28^+^) FoxP3^+^ CD8 T-cells remained unchanged in all study groups in the cross-sectional analysis, and only a significant increase was observed in acutely infected individuals in the longitudinal study (p= 0.01) (**Figure 1C**). In addition, acute HIV infection compared to uninfected controls, was linked to increased frequencies of central memory (CM, CD45RA^-^CD28^+^) (p= 0.001), effector memory (EM, CD45RA^-^CD28^-^) (p< 0.0001), and terminally differentiated (TD, CD45RA^+^CD28^-^) FoxP3^+^ CD8 T-cells (p= 0.01) (**Figures 1D-F**). Early ART initiation did not affect naïve and TD FoxP3^+^ CD8 T-cells but decreased the frequencies of both CM and EM FoxP3^+^ CD8 T-cells. Despite early ART, the frequencies of EM FoxP3^+^ CD8 T-cells remained higher than in uninfected controls (p= 0.001) (**Figure 1E**). Frequencies of CM FoxP3^+^ CD8 T-cells positively correlated with CD4 and CD8 immune activation (**Table 2**). Both CD4 and CD8 immune activation positively correlated with frequencies of EM and TD FoxP3^+^ CD8 T-cells, whereas only EM FoxP3^+^ CD8 T-cells were positively associated with VL and inversely with CD4 T-cell count and CD4/CD8 ratio (**Table 2**). ECs showed lower CM FoxP3^+^ CD8 T-cells frequencies compared to chronic (ART+) and acute (ART-), lower EM FoxP3^+^ CD8 T-cells than chronic (ART-) and acute (ART-), and lower TD FoxP3^+^ CD8 T-cells compared to chronic (ART-) (**Figures 1C, E, F**). Overall, our results showed an increased differentiation of FoxP3^+^ CD8 T-cells in acute infection that, except for EM CD8 FoxP3^+^ CD8 T-cells, was normalized by early ART.

### Early ART initiation decreased but not normalized immune activation and senescence of FoxP3^+^ CD8 T-cells

**Figure 2:**
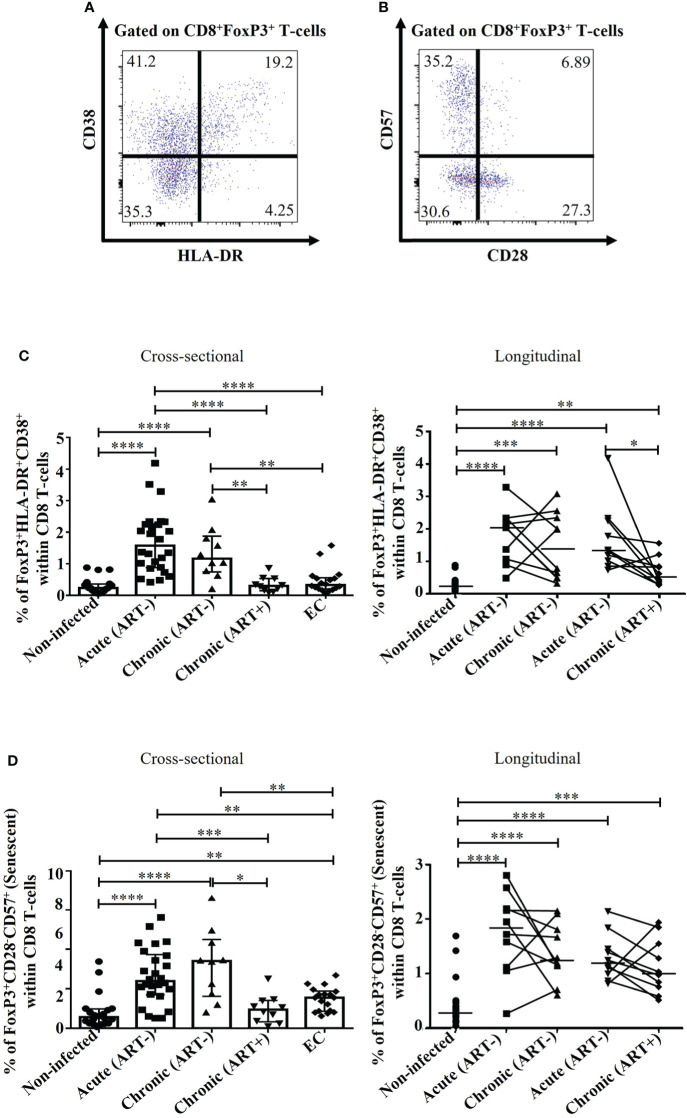
Effect of early ART initiation on FoxP3^+^ CD8 T-cell immune activation and senescence. **(A)** Gating strategies used in flow cytometry to define activated FoxP3**^+^** CD8 T-cells (CD8^+^FoxP3^+^HLA-DR^+^CD38^+^). **(B)** Gating strategies used in flow cytometry to define immunosenescent FoxP3**^+^** CD8 T-cells (CD8^+^FoxP3^+^CD28^-^CD57^+^). Frequencies of CD8^+^FoxP3^+^HLA-DR^+^CD38^+^ **(C)**, and CD8^+^FoxP3^+^CD28^-^CD57^+^ **(D)** within CD8 T-cells. Statistical significance is indicated in the figures as follow: *, P < 0.05; **, P < 0.01; ***, P < 0.001; ****, P < 0.0001. Differences among five study groups was determined by nonparametric Mann-Whitney rank test for unpaired variables, while the Wilcoxon rank tests were used for paired variables in the longitudinal study. Sample size in cross-sectional analysis: non-infected n=20, Acute n=26, Chronic ART- n=10, Chronic ART+ n=11, EC n=18. Sample size in longitudinal analysis: non-infected n=20, ART- n=10, ART+ n=10.

HIV infection was associated with increased frequencies of activated CD38^+^/HLA-DR^+^ FoxP3^+^ CD8 T-cell compared to uninfected individuals (p< 0.0001 for all comparisons in both acute (ART-) and chronic (ART-); **Figures 2A, C**). Although early ART initiation decreased FoxP3^+^ CD8 T-cell activation (CD38^+^HLA-DR^+^), it could not normalize their levels (**Figure 2C**). ECs had similar FoxP3^+^ CD8 T-cell activation than uninfected controls and showed significantly lower frequencies of activated FoxP3^+^ CD8 T-cell compared to acute and chronic ART- individuals (**Figure 2C**). HIV infection was associated with increased senescent (CD28^-^CD57^+^) FoxP3^+^ CD8 T-cells (p< 0.0001 in both cross-sectional and longitudinal analysis), while early ART initiation failed to normalize their frequencies (p=0.0001) (**Figures 2B, D**). Interestingly, ECs had lower frequencies of senescent FoxP3^+^ CD8 T-cells compared to ART- HIV-infected individuals, but these frequencies were higher than uninfected controls (p= 0.001) (**Figure 2D**). The frequency of activated and senescent FoxP3^+^ CD8 T-cells was inversely correlated with CD4 T-cell count and CD4/CD8 ratio and positively with plasma viral load and CD4 and CD8 immune activation (**Table 2**). Altogether, our results indicate that early ART initiation failed to normalize FoxP3^+^ CD8 T-cells immune activation and senescence.

### Impact of early ART initiation on immunosuppressive subsets of FoxP3^+^ CD8 T-cells

**Figure 3:**
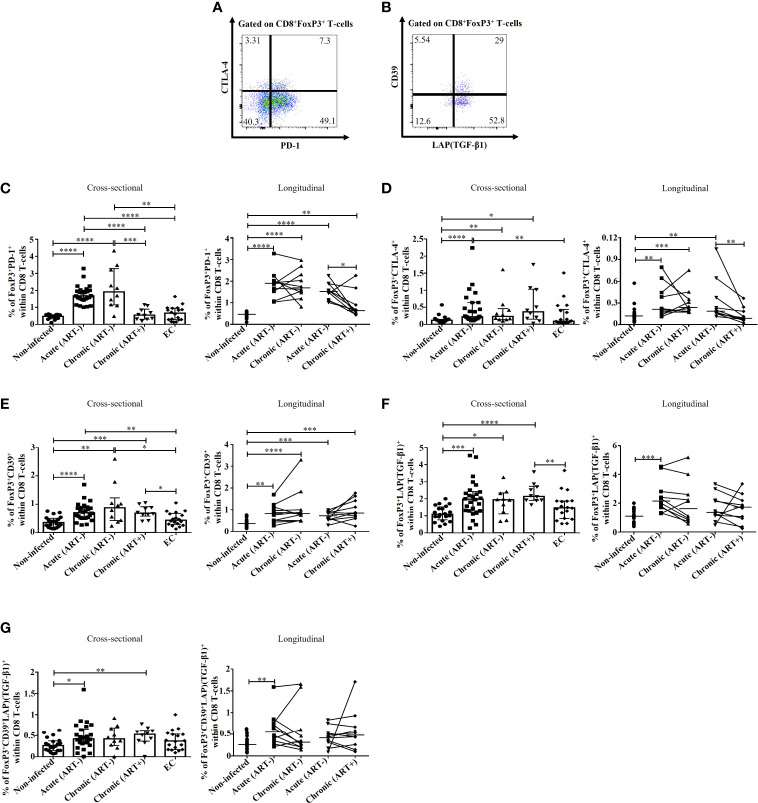
Effect of early ART initiation on FoxP3^+^ CD8 T-cells subsets with known immunosuppressive functions. Gating strategies used in flow cytometry to define FoxP3**^+^** CD8 T-cells expressing PD-1/CTLA-4 **(A)** and CD39/LAP(TGF-β1) **(B)**. Frequencies of CD8^+^FoxP3^+^PD-1^+^ **(C)**, CD8^+^FoxP3^+^CTLA-4^+^ **(D)**, CD8^+^FoxP3^+^CD39^+^ **(E)**, CD8^+^FoxP3^+^LAP(TGF-β1)^+^ **(F)**, and CD8^+^FoxP3^+^CD39^+^LAP(TGF-β1)^+^ **(G)** within CD8 T-cells. Statistical significance is indicated in the figures as follow: *, P < 0.05; **, P < 0.01; ***, P < 0.001; ****, P < 0.0001. Differences among five study groups was determined by nonparametric Mann-Whitney rank test for unpaired variables, while the Wilcoxon rank tests were used for paired variables in the longitudinal study. Sample size in cross-sectional analysis: non-infected n=20, Acute n=26, Chronic ART- n=10, Chronic ART+ n=11, EC n=18. Sample size in longitudinal analysis: non-infected n=20, ART- n=10, ART+ n=10.

As previously mentioned, FoxP3^+^ CD8 T-cells include various subsets based on the expression of PD-1 (21), CTLA-4 (16, 17, 22), CD39 (16), and TGF-β1 (12, 23, 30), which are needed for their survival and to exert immunosuppressive functions (**Figures 3A, B** **)**. HIV infection was linked to an increase in the frequencies of PD-1^+^ FoxP3^+^ CD8 T-cells (p< 0.0001 for both acute and chronic ART-) and CTLA-4^+^ FoxP3^+^ CD8 T-cells (p< 0.0001, p= 0.003 for acute and chronic ART-, respectively) compared to uninfected individuals (**Figures 3C, D**). Early ART initiation normalized CTLA-4^+^ FoxP3^+^ CD8 T-cells but not PD-1^+^ FoxP3^+^ CD8 T-cells (**Figures 3C, D**). These two populations were inversely correlated with CD4/CD8 ratio and positively with CD4 and CD8 immune activation (**Table 2**). In addition, only PD-1^+^ FoxP3^+^ CD8 T-cells negatively correlated with CD4 T-cell count and positively with VL (**Table 2**). ECs presented lower frequencies of PD-1^+^ (p< 0.0001 for both acute and chronic ART-) and CTLA-4^+^ (p= 0.009 for acute) FoxP3^+^ CD8 T-cells compared with ART- HIV-infected individuals and similar to uninfected controls (**Figures 3C, D**). HIV infection was also associated with increased frequencies of CD39^+^ FoxP3^+^ CD8 T-cells in both acute and chronic phases, and ART had no impact on their frequencies, while ECs represent similar frequencies of CD39^+^ FoxP3^+^ CD8 T-cells than uninfected controls (**Figure 3E**). CD39^+^ FoxP3^+^ CD8 T-cells frequencies correlated positively with CD4 and CD8 activation, whereas ART duration negatively correlated with this population (**Table 2**).

LAP(TGF-β1)^+^ and CD39^+^LAP(TGF-β1)^+^ FoxP3^+^ CD8 T-cell frequencies were increased in HIV acute infection compared to uninfected controls and positively correlated with CD4 and CD8 T-cell immune activation (**Figures 3F, G**; **Table 2**). Early ART initiation, but not later ART in cross-sectional analysis, inhibited their expansion (**Figures 3F, G**). Overall, our data demonstrated that early ART initiation reduced the frequencies of immunosuppressive CTLA-4^+^ FoxP3^+^ CD8 T-cells but was unable to normalize the frequencies of other immunosuppressive FoxP3^+^ CD8 T-cell subsets.

### Impact of HIV infection and early ART on migration potential of FoxP3^+^ CD8 T-cells

**Figure 4:**
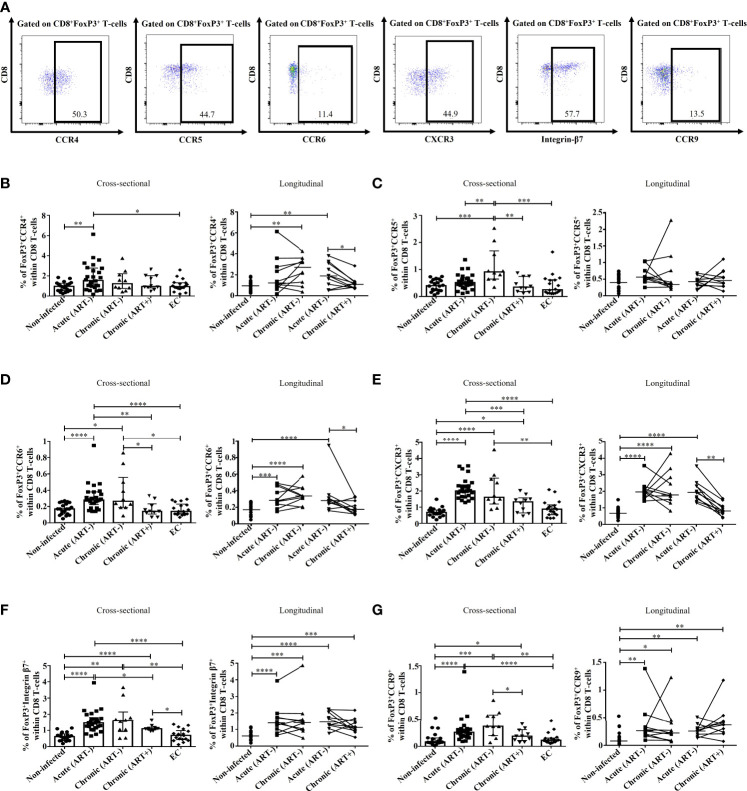
Effect of early ART initiation on FoxP3^+^ CD8 T-cells potential migration. **(A)** Gating strategies used in flow cytometry to define CD8^+^FoxP3^+^ T-cells -expressing CCR4, CCR5, CCR6, CXCR3, Integrin-β7 and CCR9. Frequencies of CD8^+^FoxP3^+^CCR4^+^ **(B)**, CD8^+^FoxP3^+^CCR5^+^ **(C)**, CD8^+^FoxP3^+^CCR6^+^ **(D)**, CD8^+^FoxP3^+^CXCR3^+^ **(E)**, CD8^+^FoxP3^+^Integrin-β7^+^ **(F)**, and CD8^+^FoxP3^+^CCR9^+^ **(G)** within CD8 T-cells. Statistical significance is indicated in the figures as follow: *, P < 0.05; **, P < 0.01; ***, P < 0.001; ****, P < 0.0001. Differences among five study groups was determined by nonparametric Mann-Whitney rank test for unpaired variables, while the Wilcoxon rank tests were used for paired variables in the longitudinal study. Sample size in cross-sectional analysis: non-infected n=20, Acute n=26, Chronic ART- n=10, Chronic ART+ n=11, EC n=18. Sample size in longitudinal analysis: non-infected n=20, ART- n=10, ART+ n=10.

We then evaluated the potential migration of FoxP3^+^ CD8 T-cells by characterizing the expression of chemokine receptors (**Figure 4A**). CCR4 binds to chemokine ligands CCL17 and CCL22 and is mainly expressed by T-cells. The expression of CCR4 has been linked to migration to the skin, heart, lung, and lymph nodes (42–45). CCR4^+^ FoxP3^+^ CD8 T-cell frequencies were higher in acute HIV infection compared to uninfected controls and ECs (p= 0.006 and p= 0.02, respectively), which was normalized by early ART initiation (**Figure 4B**). The frequencies of this population positively correlated with CD4 and CD8 immune activation (**Table 2**). CCR5 is a G-coupled receptor that binds to CCL3, CLL4, and CCL5 linked to cell migration to the brain, inflamed tissues and gut and is suggested to play an essential role in CD8 T-cells differentiation and activation (46–49). Significant increases in CCR5^+^ FoxP3^+^ CD8 T-cell frequencies were found only in the chronic ART- group, while ART+ individuals represented similar levels of CCR5^+^ FoxP3^+^ CD8 T-cells than uninfected controls and ECs (**Figure 4C**). CXCR3 is an IFN-inducible chemokine receptor that binds to chemokines CXCL4, CXCL9, CXCL10, and CXCL11, which directs the migration towards inflamed sites (50–52). We also assessed the expression of CCR6 (53, 54), CCR9 (55–57), and Integrin-β7 (53, 57), which direct T-cells recruitment towards the gut through the binding of CCL20, CCL25, and Mucosal vascular-Addressin Cell-Adhesin Molecule 1, respectively. Frequencies of CCR6^+^ and CXCR3^+^ FoxP3^+^ CD8 T-cells were increased in untreated acute and chronic ART- HIV infection (**Figures 4D, E**). Similarly, FoxP3^+^ CD8 T-cells expressing gut homing markers CCR9 and Integrin-β7 were also rapidly increased in acute and chronic ART- infection. However, in contrast to CCR6 and CXCR3, early ART initiation was unable to normalize the levels of CCR9^+^ and Integrin-β7^+^ FoxP3^+^ CD8 T-cells (**Figures 4F, G** **)**. ECs have a similar expression of these chemokine receptors than uninfected controls (**Figures 4F, G** **)**. The frequencies of CCR6^+^, CXCR3^+^, CCR9^+^, and Integrin-β7^+^ FoxP3^+^ CD8 T-cells were negatively correlated with CD4 T-cell count and CD4/CD8 ratio, while positively correlated with T-cell immune activation and plasma VL (**Table 2**). Moreover, only CCR9^+^ FoxP3^+^ CD8 T-cells inversely correlated with the duration of ART (**Table 2**).

**Figure 5:**
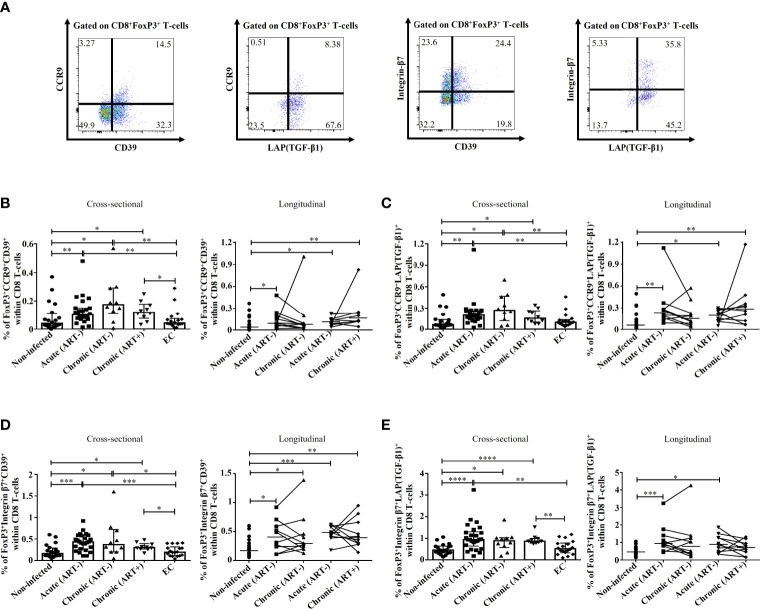
Effect of early ART initiation on the migratory potential towards the gut of FoxP3^+^ CD8 T-cells subsets with known immunosuppressive functions. **(A)** Gating strategies used in flow cytometry to define CCR9^+^CD39^+^, CCR9^+^LAP(TGF-β1)^+^, Integrin-β7^+^CD39^+^, and Integrin-β7^+^LAP(TGF-β1)^+^ CD8^+^FoxP3^+^ T-cells. Frequencies of CD8^+^FoxP3^+^CCR9^+^CD39^+^ **(B)**, CD8^+^FoxP3^+^CCR9^+^LAP(TGF-β1)^+^ **(C)**, CD8^+^FoxP3^+^Integrin-β7^+^CD39^+^ **(D)**, and CD8^+^FoxP3^+^Integrin-β7^+^LAP(TGF-β1)^+^ **(E)** within CD8 T-cells. Statistical significance is indicated in the figures as follow: *, P < 0.05; **, P < 0.01; ***, P < 0.001; ****, P < 0.0001. Differences among five study groups was determined by nonparametric Mann-Whitney rank test for unpaired variables, while the Wilcoxon rank tests were used for paired variables in the longitudinal study. Sample size in cross-sectional analysis: non-infected n=20, Acute n=26, Chronic ART- n=10, Chronic ART+ n=11, EC n=18. Sample size in longitudinal analysis: non-infected n=20, ART- n=10, ART+ n=10.

We also assessed the potential migration of immunosuppressive CD39^+^ and LAP(TGF-β1)^+^ FoxP3^+^ CD8 T-cells towards the gut by their expression of CCR9 and Integrin-β7. Here again, we observed that untreated HIV infection was associated with increases in CD39^+^ and LAP(TGF-β1)^+^ FoxP3^+^ CD8 T-cells expressing CCR9 and Integrin-β7 compared to ECs and uninfected controls (**Figure 5**). Importantly, early ART initiation failed to normalize the gut migration potential of these subsets expect for Integrin-β7^+^LAP(TGF-β1)^+^ FoxP3^+^ CD8 T-cells. The frequencies of CD39^+^ and LAP(TGF-β1)^+^ FoxP3^+^ CD8 T-cells expressing CCR9 and Integrin-β7 were all positively correlated with T-cell immune activation, and, excepting Integrin β7^+^LAP(TGF-β1)^+^, they negatively correlated with longer duration of ART (**Table 2**). Furthermore, we found a positive correlation between VL and Integrin β7^+^CD39^+^ FoxP3^+^ CD8 T-cells frequencies, whereas Integrin β7^+^LAP(TGF-β1)^+^ FoxP3^+^ CD8 T-cells were negatively correlated with CD4/CD8 ratio (**Table 2**). Altogether, our results showed that during HIV infection and despite early ART initiation, immunosuppressive CD39^+^ and LAP(TGF-β1)^+^ FoxP3^+^ CD8 T-cells maintained their capacity to migrate to the gut, which, in turn, could contribute to gut mucosal immune dysfunction and tissue fibrosis.

### Early ART was unable to normalize CD28^-^PD-1^+^ and CD28^-^CD39^+^ CD8 T-cell subsets

**Figure 6:**
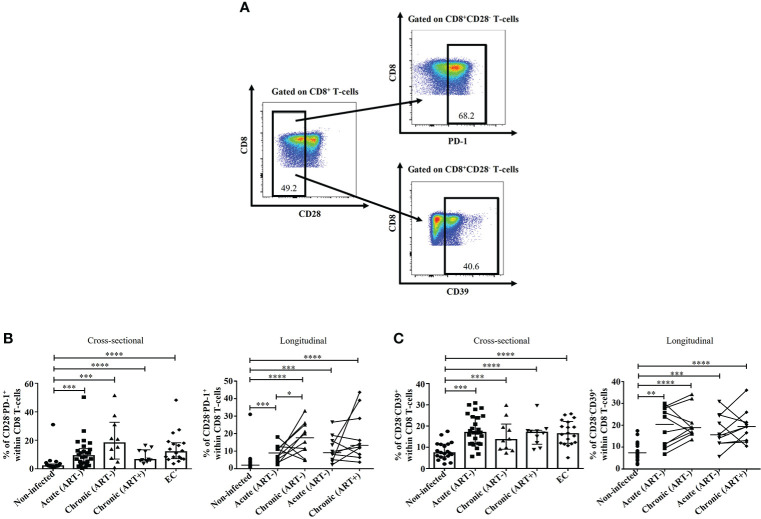
Effect of early ART initiation on CD8^+^CD28^-^PD-1^+^ and CD8^+^CD28^-^CD39^+^ T-cells. Gating strategies used in flow cytometry to define CD8^+^CD28^-^PD-1^+^ and CD8^+^CD28^-^CD39^+^ **(A)**. Frequencies of CD8^+^CD28^-^PD-1^+^ **(B)** and CD8^+^CD28^-^CD39^+^ **(C)** within CD8 T-cells. Statistical significance is indicated in the figures as follow: *, P < 0.05; **, P < 0.01; ***, P < 0.001; ****, P < 0.0001. Differences among five study groups was determined by nonparametric Mann-Whitney rank test for unpaired variables, while the Wilcoxon rank tests were used for paired variables in the longitudinal study. Sample size in cross-sectional analysis: non-infected n=20, Acute n=26, Chronic ART- n=10, Chronic ART+ n=11, EC n=18. Sample size in longitudinal analysis: non-infected n=20, ART- n=10, ART+ n=10.

In addition to classical FoxP3^+^ CD8 T-cells, other CD8 T-cell subsets have also been described as immunosuppressive regardless of their FoxP3 expression, including CD8^+^CD28^-^PD-1^+^ and CD8^+^CD28^-^CD39^+^ CD8 T-cells (7, 8, 28, 58). Herein, we observed a rapid expansion of both CD28^-^PD-1^+^ and CD28^-^CD39^+^ CD8 T-cell subsets in untreated HIV infection compared to ECs and uninfected controls (**Figure 6**). Importantly, their frequencies were not affected by early ART initiation. Both populations correlated positively with CD4 and CD8 immune activation, while only CD28^-^PD-1^+^ negatively correlated with CD4/CD8 ratio, and only CD28^-^CD39^+^ negatively correlated with the duration of ART (**Table 2**).

## Discussion

The immune responses exerted by effector CD8 T-cells are crucial for controlling SIV/HIV infections (59, 60). In contrast, immunosuppressive functions of FoxP3^+^ CD8 T-cells are primarily detrimental since higher frequencies of these cells have been associated with immune dysfunction, viral persistence and HIV disease progression (15–17). We recently showed that early ART initiation in HIV-infected individuals was unable to reduce CD39^+^ and LAP(TGF-β1)^+^ CD4 Tregs and their potential migration to the gut (40). Herein, in the same study cohort, we showed that acute HIV infection increased the frequencies of FoxP3^+^ CD8 T-cells, which were normalized by early ART initiation. Importantly, although we observed an overall increase in FoxP3 expression on CD8 T-cells in untreated HIV infection that could affect the relative proportions of FoxP3^+^ subsets reported in our study, we also observed clear differences between the expression of various markers on FoxP3^+^ CD8 T-cells *versus* total CD8 T-cells which suggest the particular dynamics of FoxP3^+^ CD8 T-cells during HIV infection (**Supplementary Table 2**). In contrast, early treatment was unable to normalize FoxP3^+^ CD8 T-cell activation and senescence, as well as the gut migratory potential of CD39^+^ and LAP(TGF-β1)^+^ FoxP3^+^ CD8 T-cells.

Similar to previous studies in both human and RMs, we observed increased frequencies of total FoxP3^+^ CD8 T-cells in untreated HIV-infected individuals (15–17, 23). Notably, the reduction in FoxP3^+^ CD8 T-cells following early ART initiation was reported in SIV-infected RMs (38, 39), while no studies in humans, to the best of our knowledge, have evaluated its effect on HIV-infected individuals. ECs showed lower FoxP3^+^ CD8 T-cell frequencies than HIV-progressors and were comparable to uninfected individuals, which contrasts with a unique report of an increase in FoxP3^+^ CD8 T-cells in SIV controllers Indian RMs compared to SIV progressor monkeys (18). These differences could be associated with increased viral fitness and VL and faster disease progression in the Indian RM model (61). In our study, the EC group is significantly older and with a longer duration of the infection, which can also impact our observations. In line with previous reports, our results showed that total FoxP3^+^ CD8 T-cell frequencies were linked to markers of disease progression such as CD4 T-cell count, CD4/CD8 ratio, and VL (17).

Untreated HIV infection was associated with an early increase in FoxP3^+^ CD8 T-cell immune activation (HLA-DR^+^/CD38^+^) and senescence (CD28^-^CD57^+^) that remained elevated despite early ART initiation. The maintenance of activated FoxP3^+^ CD8 T-cells following early ART is significantly important since activated CD8 T-cells have higher proliferation (60), and HLA-DR^+^ CD8 T-cells are highly immunosuppressive comparable to CD4 Tregs (62). Even in the absence of viremia under successful ART, immune activation persists and promotes immunosenescence (63), which could explain higher levels of immunosenescent FoxP3^+^ CD8 T-cells regardless of early ART initiation. The increase in immunosenescent FoxP3^+^ CD8 T-cells in ECs compared to uninfected individuals, while having similar levels of immune activation, could be related to the age of these individuals since a positive correlation between age and CD28^-^CD57^+^ CD8 T-cells was only observed in ECs (data not shown) (64).

A distinctive differentiation pattern of FoxP3^+^ CD8 T-cells was observed in untreated HIV infection, characterized by an increase in CM, EM, and TD FoxP3^+^ CD8 T-cells that, excepting for EM FoxP3^+^ CD8 T-cells, were normalized by early ART, indicating an increased differentiation of FoxP3^+^ CD8 T-cells in HIV infection. The increase in total FoxP3^+^ CD8 T-cells and their differentiation could be related to the expansion of antigen-experienced FoxP3^+^ CD8 T-cells or conversion of antigen-primmed FoxP3^-^CD8 T-cells into FoxP3^+^ CD8 T-cells by TGF-β1 (65). The persistence of higher frequencies of EM FoxP3^+^ CD8 T-cells despite early ART is particularly important. In fact, EM T-cells show an increased ability to localize within tissues and migrate into non-lymphoid tissues in response to infection or inflammation (66, 67), suggesting a higher FoxP3^+^ CD8 T-cell migratory potential towards inflammatory sites and the gut. In this regard, we observed increased frequencies of FoxP3^+^ CD8 T-cells expressing migration markers to inflammatory sites and the gut and the persistence of CCR9/Integrin β7 FoxP3^+^ CD8 T-cells despite early ART. Moreover, the increase in highly differentiated FoxP3^+^CD8 T-cells is in line with the increase in CCR5^+^ FoxP3^+^ CD8 T-cells since CCR5 expression is associated with an increase in CD8 functions and differentiation (49, 68, 69). We also observed increased CCR4^+^ and CXCR3^+^ FoxP3^+^ CD8 T-cells in untreated HIV infection, which was restored by early ART initiation. Importantly, CCR4 expression is associated with higher CD4 Tregs inhibitory capacity and could have similar functions in FoxP3^+^ CD8 T-cells (70), while anti-CCR4 treatment decreases CD8 T-cell immune responses (71). On the other hand, CXCR3^+^ CD8 T-cells are well-known IL-10 producers immunosuppressor cells (72), and CXCR3 expression is a reliable marker for EM CD8 T-cells immune responses (73). In addition, CXCR3 expression regulates CD8 T-cells differentiation in acute and chronic viral infections (74). FoxP3^+^ CD8 T-cells could co-localize with CD4 T-cells expressing similar chemokine receptors and further inhibit their proliferation and anti-HIV-specific response. Importantly, CCR5^+^ and CCR6^+^ CD4 T-cells are highly susceptible to HIV infection (75–77). Thus, FoxP3^+^ CD8 T-cell colocalization with CD4 T-cells mediated by CCR5 and CCR6-dependent recruitment could contribute to poor viral control and disease progression. Moreover, a model of colocalization between HIV-specific CD8 and CD4 T-cells in the gut pointed to integrin β7 rather than CCR6 as the mediator of this migration (53).

An increase in various immunosuppressive subsets of FoxP3^+^ CD8 T-cells, including CTLA-4^+^, PD-1^+^, CD39^+^, LAP(TGF-β1)^+^, and CD39^+^LAP(TGF-β1)^+^ was observed in untreated HIV infection, whereas early ART initiation was unable to normalize levels of PD-1^+^, and did not affect CD39^+^ and LAP(TGF-β1)^+^FoxP3^+^ CD8 T-cells. Similarly, in the same study cohort, we also observed that early ART initiation failed to normalize PD-1^+^ and CD39^+^ CD4 Tregs (40). However, we recently reported that very early ART initiation at four days post-SIV infection of RMs reduced the frequencies of CD39^+^ FoxP3^+^ CD8 T-cells (39). Furthermore, longer ART treatment correlated negatively with CD39^+^ FoxP3^+^ CD8 T-cell frequencies, which could indicate that earlier ART initiation and longer treatment contribute to better control of their expansion. Despite early ART initiation, the persistence of PD-1^+^ and CD39^+^ FoxP3^+^ CD8 T-cells could contribute to immune dysfunction and disease progression. Indeed, PD-1/PD-1L contributes to FoxP3^+^ CD8 T-cells immunosuppression by increasing FoxP3^+^ CD8 T-cells proliferation/differentiation and inducing apoptosis in effector cells (21, 62). Importantly, PD-1/PD-1L interaction induces FoxP3 expression and promotes CD4 Tregs expansion (78–80). Thus, it is logical to think that a similar process might occur in CD8 T-cells promoting FoxP3 stabilization and FoxP3^+^ CD8 T-cells expansion. Furthermore, the increase in LAP(TGF-β1)^+^ FoxP3^+^ CD8 T-cells during untreated infection is supported by a report of a positive correlation between TGF-β1 production and FoxP3^+^ CD8 T-cells frequencies in non-pathogenic SIV infection in African green monkeys (23). Moreover, downstream genes of the TGF-β1 pathway are upregulated as early as one day after SIV infection in RMs (81) and HIV-infected individuals (82). FoxP3^+^ CD8 T-cells in ECs expressed similar CTLA-4, PD-1, CD39, and LAP(TGF-β1) levels than uninfected individuals, which could be associated with the maintenance of effector cell functions and viral control in these individuals.

Similar to our recent report on CD4 Tregs (40), we observed increased CCR9^+^ and integrin β7^+^ FoxP3^+^ CD8 T-cells, along with CD39^+^ and LAP(TGF-β1)^+^ FoxP3^+^ CD8 T-cells during untreated HIV infection that persisted regardless of early ART initiation. These findings are particularly significant, suggesting a potential migration of FoxP3^+^ CD8 T-cells with known immunosuppressive potential towards the gut despite ART. Interestingly, CD4 Tregs can promote the proliferation of FoxP3^+^ CD8 T-cells and vice versa. Indeed, each cell type’s IL-10 and TGF-β1 may contribute to FoxP3 expression and differentiation of the other subset (12, 29). Moreover, both CD4^+^ and CD8^+^FoxP3^+^ T-cells have previously been shown to work together in animal models, where the participation of both Treg subsets is significantly higher in combined transfers than in independent transfers (13, 83, 84). The migration of highly immunosuppressive CD39^+^ and LAP(TGF-β1)^+^ FoxP3^+^ CD8 T-cells to the gut could inhibit specific antiviral responses while promoting immune dysfunction and tissue fibrosis (16, 34). Notably, functional interplays between CD39 and TGF-β1 are also known. Indeed, TGF-β1 production, tissue remodeling, and fibrosis are promoted by CD39 enzymatic activity and adenosine production (85–87), whereas TGF-β1 signaling stimulates CD39 expression and activity (88–91). Moreover, an increase in TGF-β1 production and activity by the adenosine pathway may also stimulate FoxP3^+^ Tregs expansion (92, 93). Interestingly, TGF-β1 upregulates CTLA-4 and PD-1 expression (94), and we observed an increase in the expression of both markers. This indicates that in addition to promoting fibrosis and inducing CD39 and FoxP3 expression, TGF-β1 can also contribute to immunosuppression by inducing immune checkpoints PD-1 and CTLA-4. Notably, total CCR9^+^ FoxP3^+^ CD8 T-cells and CD39^+^/LAP(TGF-β1)^+^ FoxP3^+^ CD8 T-cells expressing CCR9 remained elevated despite early ART initiation, but their frequencies were negatively correlated with the duration of ART, suggesting that longer ART duration rather than earlier interventions could decrease their frequencies.

Finally, we observed an increase in both CD28^-^PD-1^+^ and CD28^-^CD39^+^ CD8 T-cells - two subsets with immunosuppressive functions regardless of FoxP3 expression - which was not restored following early ART initiation. The increase in CD28^-^PD-1^+^ (15, 95) and CD28^-^CD39^+^ (28) CD8 T-cells in untreated HIV-infected individuals correspond with previous reports. CD28^-^ CD8 T-cells are known to induce tolerogenic dendritic cells and secretion of inhibitory cytokines such as IL-10 and TGF-β1 (96). CD28^-^PD-1^+^ phenotype is associated with immune exhaustion, poor anti-HIV specific response, and disease progression (97, 98). Thus, increased CD28^-^PD-1^+^ CD8 T-cells during untreated HIV infection and their persistence regardless of ART indicates exhaustion and potentially dysfunctionality of CD8 T-cells despite early ART. Moreover, CD28^-^CD39^+^ CD8 T-cells could contribute to immune dysfunction and disease progression through similar mechanisms than FoxP3^+^CD39^+^ CD8 T-cells.

Our study had some limitations which deserve to be discussed, including the relatively small sample size. Nevertheless, our findings were consistent with previous findings using a similar sample size and had a high biological plausibility (40, 99, 100). We recognize that variables such as gender, age, duration of infection, and timing of ART initiation may influence our findings. In this regard, the expression of FoxP3 and other Treg markers can be influenced by sex hormones and gender (101, 102). Similarly, the frequencies and functions of FoxP3^+^ CD8 T-cells and the expression of immunosuppressive markers by these cells differ in older individuals (103, 104). We mainly recruited male participants in our analysis since they constitute the majority of the participants in the Montreal primary HIV infection (acute) cohort. Additionally, we did not provide functional assays to assess FoxP3^+^ CD8 T-cells’ inhibitory capacity since we had limited access to these specimens and the fact that FACS-sorting of FoxP3^+^ CD8 T-cells requires the permeabilization and fixation of the cells which are not usable for *in vitro* functional assays. Ultimately, while we used well-established markers of T-cell migration to the gut, all analyses were performed in peripheral blood as an indirect indication of FoxP3^+^ CD8 T-cell migration towards this compartment.

In summary, for the first time, we spotlight various subsets of FoxP3^+^ CD8 T-cells that might be critical in HIV disease progression. We showed that early ART initiation did not normalize the frequency of immunosuppressive and pro-fibrogenic FoxP3^+^ CD8 T-cells and their potential migration to the gut. The latter can contribute to immune dysfunction, gut fibrosis, and HIV disease progression, suggesting that other therapies combined with early ART initiation are needed to reduce FoxP3^+^ CD8 T-cells immunosuppressive subsets.

## Data availability statement

The original contributions presented in the study are included in the article/**supplementary material**. Further inquiries can be directed to the corresponding author.

## Ethics statement

The studies involving human participants were reviewed and approved by the ethical review board of the Université du Québec à Montréal (UQAM) gave their approval to this study (#2014-452), which followed the Declaration of Helsinki. All study participants signed a written informed consent form before blood collection. The patients/participants provided their written informed consent to participate in this study.

## Author contributions

M-AJ designed the study. AY and TS performed the experiments. J-PR, CT, and CC provided access to specimens and clinical data. AY, TS, MD, CC, and M-AJ analysed, discussed, and interpreted results throughout the study. AY and M-AJ wrote the paper. All authors contributed to the refinement of the study and reviewed and approved the final version of manuscript.

## Funding

This study was funded by the Canadian Institutes of Health Research (CIHR, grant MOP 142294) and in part by the AIDS and Infectious Diseases Network of the Réseau SIDA et maladies infectieuses du Fonds de recherche du Québec-Santé (FRQ-S) to M-AJ. AY is the recipient of an FRQ-S doctorate fellowship. J-PR is the Louis Lowenstein Chair in Hematology and Oncology at McGill University. CT is the Pfizer Chair in Clinical and Translational HIV Research. MD and CC are recipients of FRQ-S Junior 2 Clinician-Researcher career awards. M-AJ holds the tier 2 CIHR Canada Research Chair in Immuno-Virology. The funding institutions had no involvement in the data collection, analysis, or interpretation.

## Acknowledgments

First and foremost, the authors like to express their gratitude to all study participants for their time and commitment to this research. Individuals in acute infection were screened, recruited, and followed up at the following Montreal-based clinics: l’Actuel Medical Clinic, Quartier Latin Medical Clinic, UHRESS CHUM Hôtel-Dieu, and Notre-Dame, and MUHC Chest Institute. We gratefully acknowledge their assistance. Mr. Mario Legault provided administrative help, Ms. Angie Massicotte provided assistance and coordination, and Ms. Stephanie Matte assisted us with the Canadian HIV Slow Progressor cohort.

## Conflict of interest

The authors declare that the research was conducted in the absence of any commercial or financial relationships that could be construed as a potential conflict of interest.

## Publisher’s note

All claims expressed in this article are solely those of the authors and do not necessarily represent those of their affiliated organizations, or those of the publisher, the editors and the reviewers. Any product that may be evaluated in this article, or claim that may be made by its manufacturer, is not guaranteed or endorsed by the publisher.
