# Supplementary material for: FoxP3+ CD8 T-cells in acute HIV infection and following early antiretroviral therapy initiation
Source: Front Immunol. 2022 Jul 29;13:962912. doi: 10.3389/fimmu.2022.962912 (PMC9372390; doi:10.3389/fimmu.2022.962912)
Supplement: Supplementary file 1 [file Table_1.docx]

| **mAb-Fluorochrome** | **Clone** | **Company** | **Catalog** **number** |
| --- | --- | --- | --- |
| CD152 (CTLA-4)-APC | BNI3 | BD Pharmingen™ | 555855 |
| CD183 (CXCR3)-PEcy5 | 1C6/CXCR3 | BD Pharmingen™ | 551128 |
| CD194 (CCR4)-BV421 | 1G1 | BD Horizon™ | 562579 |
| CD195 (CCR5)-BV605 | 2D7/CCR5 | BD Horizon™ | 563379 |
| CD196 (CCR6)-BB515 | 11A9 | BD Horizon™ | 564479 |
| CD199 (CCR9)-APC | L053E8 | BioLegend | 358908 |
| CD279 (PD-1)-BV711 | EH12.2H7 | BioLegend | 329928 |
| CD28-PEcy5 | CD28.2 | BD Pharmingen™ | 555730 |
| CD38-PEcy7 | HIT2 | BD Pharmingen™ | 560677 |
| CD39-BV711 | TU66 | BD Horizon™ | 563680 |
| CD3-BV786 | UCHT1 | BD Horizon™ | 565491 |
| CD45RA-BV650 | HI100 | BD Horizon™ | 563963 |
| CD4-APC-H7 | RPA-T4 | BD Pharmingen™ | 560158 |
| CD4-FITC | RPA-T4 | BD Pharmingen™ | 555346 |
| CD57-APC | NK-1 | BD Pharmingen™ | 560845 |
| CD8-Alexa Fluor 700 | RPA-T8 | BD Horizon™ | 561453 |
| FoxP3-PE CF594 | 236A/E7 | BD Horizon™ | 563955 |
| HLA-DR-BV605 | G46-6 | BD Horizon™ | 562845 |
| Integrin β7-FITC | FIB504 | BioLegend | 321212 |
| LAP(TGF-β1)-BV421 | TW4-2F8 | BioLegend | 349613 |

**Supplementary Table 1**. List of mAbs used for flow cytometry.
