# Supplementary material for: FoxP3+ CD8 T-cells in acute HIV infection and following early antiretroviral therapy initiation
Source: Front Immunol. 2022 Jul 29;13:962912. doi: 10.3389/fimmu.2022.962912 (PMC9372390; doi:10.3389/fimmu.2022.962912)
Supplement: Supplementary file 2 [file Table_2.docx]

|  | **Non-infected** | | **Acute (ART-)** | | **Chronic (ART-)** | | **Chronic (ART+)** | | **Elite controllers** | |
| --- | --- | --- | --- | --- | --- | --- | --- | --- | --- | --- |
|  | **% within total CD8^+^** | **% within FoxP3^+^ CD8^+^** | **% within total CD8^+^** | **% within FoxP3^+^ CD8^+^** | **% within total CD8^+^** | **% within FoxP3^+^ CD8^+^** | **% within total CD8^+^** | **% within FoxP3^+^ CD8^+^** | **% within total CD8^+^** | **% within FoxP3^+^ CD8^+^** |
| **Naïve (CD45RA^+^CD28^+^)**  **median (IQR)** | 23.65^a,b,c,d,k^  (17.38-28.40) | 8.13^k^  (6.41-13.7) | 10.03^a,l^  (6.25-19.65) | 6.56^l^  (3.77-10.38) | 11.80^b,m^  (9.13-21.35) | 7.95^g,m^  (2.66-15.48) | 13.40^c^  (9.55-19.78) | 13.50  (3.89-27.95) | 14.85^d^  (7.24-24.03) | 13.35^g^  (7.12-19.08) |
| **CM (CD45RA^-^CD28^+^)**  **median (IQR)** | 22.50^b,d^  (594-36.43) | 23.55  (16.75-36.30) | 18.55^l^  (13.75-27.60) | 19.35^l^  (17.20-31.63) | 13.95^b,h^  (11.38-16.98) | 17.15^h^  (10.03-21.68) | 28.85^h,j^  (15-48-55) | 33^h,j^  (20.73-52.85) | 17.55^d,j,o^  (8.32-21.10) | 18.40^j,o^  (10.83-31.50) |
| **EM (CD45RA^-^CD28^-^)**  **median (IQR)** | 11.15^a,b,d,k^  (5.38-16.28) | 22.45^a,k^  (13.43-27.43) | 33.65^a,f,g,l^  (22-42.63) | 35.45^a,f,g,l^  (27.10-47.38) | 29.95^b,h,i^  (24.93-36.18) | 32.60  (18.95-45.43) | 14.05^f,h^  (9.37-16.55) | 18.60^f^  (8.64-33.75) | 19.30^d,g,i^  (7.93-32.03) | 18.80^g^  (9.31-30.90) |
| **TD (CD45RA^+^CD28^-^)**  **median (IQR)** | 40.50^c^  (26-52.35) | 40.70  (25.05-53.73) | 34.80^g^  (23.33-44.78) | 30.45^g^  (21.25-41.13) | 39.70  (33.90-44.60) | 38.05  (29.25-48.38) | 26.40^j^  (13.80-52.35) | 19.60^c,j^  (11.33-44.45) | 42.10^g,j^  (38.85-58.35) | 40^g,j^  (31.33-56.63) |
| **CD38^+^HLA-DR^+^**  **median (IQR)** | 3.32^a,b,c,d,k^  (1.44-4.98) | 19.35^a,b,k^  (13.80-26.23) | 26.15^a,f ,g,l^  (18.20-34.40) | 39.45^a,f,g,l^  (23.95-58.48) | 32.20^b,h,I,m^  (26.25-38.65) | 38.95^b,h,I,m^  (30.53-50.13) | 10.17^c,f,h,n^  (7.97-15.85) | 18.55^f ,h,n^  (15.93-30.68) | 11.75^d,g,I,o^  (4.68-21.58) | 24.90^g,I,o^  (18.25-34.70) |
| **CD57^+^CD28^-^**  **median (IQR)** | 15.45^a,b,d^  (6.72-26.75) | 13.40^a,b,d^  (8.32-29.65) | 40.20^a,f,l^  (23.75-47.58) | 34.45^a,f,l^  (19.43-40.73) | 34.25^b^  (25.28-48.88) | 27.85^b,h^  (22.65-46.60) | 23.15^f,n^  (10.40-31.05) | 18.40^f,h,j,n^  (12.11-25.40) | 32.85^d^  (20.85-45.30) | 31.55^d,j^  (23.18-43.48) |
| **PD-1^+^**  **median (IQR)** | 7.97^a,b,c,d,k^  (5.44-19.68) | 28.50^a,b,k^  (23.23-31.60) | 30.40^a,f,g,l^  (20.55-44.23) | 40.70^a,f,g,l^  (34.08-52.30) | 34.35^b,h,m^  (27.15-47.53) | 44^b,h,I,m^  (35.18-44.05) | 19.65^c,f,h,n^  (14.53-24.10) | 21.30^f,h,n^  (17.88-39.85) | 16.90^d,g,o^  (11.32-34.88) | 31.45^g,I,o^  (17-40.33) |
| **CTLA-4^+^**  **median (IQR)** | 1.19^a,c,k^  (0.82-1.56) | 4.19^c,k^  (4.88-9.73) | 1.86^a,l^  (1.27-2.66) | 6.92^f,l^  (3.96-12.73) | 1.82^m^  (1.15-2.98) | 5.62^h,m^  (4.36-6.49) | 1.97^c,n^  (1.32-5.04) | 14.65^c,f,h,n^  (7.55-52.25) | 1.61^o^  (0.77-5.96) | 6.83^o^  (3.94-20.15) |
| **CD39^+^**  **median (IQR)** | 5.26^a,c,d,k^  (2.29-7.69) | 24.85^k^  (14.80-34.88) | 16.28^a,e,l^  (9.13-23.58) | 23.25^l^  (17.25-31.03) | 7.84^e,h,I,m^  (3.73-13.35) | 21.35^m^  (15.25-29.90) | 17.92^c,h,n^  (9.99-21.74) | 24.77^n^  (17.88-30.58) | 14.60^d,I,o^  (12.05-20.63) | 24.73^o^  (20.28-27.90) |
| **LAP(TGF-β1)^+^**  **median (IQR)** | 6.82^k^  (2.81-19) | 87.50^a,b,k^  (59.05-94.98) | 5.27^f,g,l^  (3.47-8.55) | 63.30^a,e,f,g,l^  (50.35-79.80) | 6.04^h,m^  (3.31-7.81) | 49.97^b,e,h,I,m^  (31.18-56.50) | 12.25^f,h,n^  (9.87-23.48) | 81.40^f,h,n^  (63.17-95.33) | 11.90^g,o^  (5.80-19.88) | 81.20^g,I,o^  (66.05-95.35) |
| **CD39^+^LAP(TGF-β1)^+^**  **median (IQR)** | 0.54^c,d,k^  (0.17-1.29) | 16.90^b,k^  (11.80-26.75) | 1.075^f,l^  (0.49-1.52) | 14.86^l^  (9.16-21.08) | 0.76^h,m^  (0.24-1.37) | 11.96^b,h,I,m^  (6.78-13.60) | 2.13^c,f,h,n^  (1.77-2.56) | 16.93^h,n^  (14.15-20.92) | 1.97^d,o^  (0.73-3.60) | 18.80^i,o^  (13.13-26) |
| **CCR4^+^**  **median (IQR)** | 20.60^a,k^  (10.95-33.33) | 68.80^a,b,k^  (41.70-77.80) | 10.30^a,f,g,l^  (7.36-22) | 44.60^a,l^  (21.90-62.63) | 19.05^m^  (6.96-26.93) | 29.55^b,h,I,m^  (18.58-44.73) | 23.65^f,n^  (21.45-33.3) | 62.60^h,n^  (45.73-69.70) | 19.20^g,o^  (11.95-30.48) | 53.60^i,o^  (37.30-70) |
| **CCR5^+^**  **median (IQR)** | 8.51^b,k^  (4.61-14.78) | 22.60^a,k^  (14.05-33.63) | 5.09^e,l^  (3.24-8.81) | 11.85^a,e,l^  (10.45-15.18) | 13.80^b,e,h,I,m^  (10.53-18.28) | 21.70^e,m^  (17.05-28.88) | 5.30^h,n^  (3.58-8.20) | 15.70^n^  (11.42-35.63) | 7.63^i,o^  (4.16-9.57) | 14.90^o^  (11.78-31.03) |
| **CCR6^+^**  **median (IQR)** | 7.28^a,c,d,k^  (5.25-8.44) | 10.50^a,b,c,k,d^  (8.02-13.48) | 5.11^a,l^  (3.99-6.68) | 7.25^a,l^  (5.49-8.40) | 5.36^m^  (3.94-7.66) | 7.70^b,m^  (6.90-9.75) | 4.69^c,n^  (4.43-5.63) | 6.84^c,n^  (5.71-7.58) | 5.75^d,o^  (4.01-7.58) | 7.85^d,o^  (7.23-9.79) |
| **CXCR3^+^**  **median (IQR)** | 16.85^d,k^  (10.90-22.60) | 47.05^a,k^  (35.95-52.42) | 17.75^g,l^  (9.90-26.63) | 54.90^a,l^  (39.33-68.35) | 11.35^i,m^  (9.99-13.03) | 40.50^m^  (34.90-53.33) | 14.10^j,n^  (34.90-53.33) | 52.75^n^  (43.78-62.18) | 8.02^d,g,I,j,o^  (5.58-12.30) | 45.25^o^  (36.93-59.05) |
| **CCR9^+^**  **median (IQR)** | 0.89^a,b,c,d,k^  (0.52-1.65) | 6.43^k^  (3.92-8.65) | 2.67^a,l^  (1.87-3.75) | 8.18^l^  (6.60-11.73) | 3.15^b,m^  (2.42-3.63) | 8.89^m^  (6.49-11.73) | 3.12^c,n^  (1.87-3.25) | 6.17^n^  (4.16-10.01) | 1.90^d,o^  (1.09-3.38) | 7.31^o^  (5.82-8.57) |
| **Integrin β7+**  **median (IQR)** | 6.14^a,b,c,d,k^  (4.23-7.34) | 38.60^a,k^  (33.75-52.25) | 30.70^a,l^  (21.88-40.38) | 47.99^a,e,f,g,l^  (42.63-57.18) | 29.40^b,m^  (22.10-35.53) | 38.71^e,m^  (30-47.35) | 30.50^c,n^  (24-32.21) | 39.20^f,n^  (36.13-42.98) | 26.95^d,o^  (17.18-42.40) | 35.85^g,o^  (26.18-46.78) |
| **CCR9^+^CD39^+^**  **median (IQR)** | 0.12^a,b,c,d,k^  (0.05-0.22) | 2.95^k^  (1.50-6.40) | 0.54^a,l^  (0.26-0.87) | 3.23^l^  (2.34-5.95) | 0.48^b,m^  (0.13-0.90) | 4.34^m^  (3.60-5.25) | 0.50^c,n^  (0.24-1.01) | 4^n^  (3.14-5.81) | 0.18^d,o^  (0.13-0.98) | 3.01^o^  (1.97-3.88) |
| **CCR9^+^LAP(TGF-β1)^+^**  **median (IQR)** | 0.28^a,b,c,k^  (0.09-0.54) | 4.29^k^  (3.26-8.09) | 0.61^a,l^  (0.31-0.77) | 6.56^l^  (4.73-8.50) | 0.57^b,m^  (0.27-1.19) | 6.02^m^  (3.14-10.83) | 0.93^c,n^  (0.49-1.14) | 3.55^n^  (2.52-5.68) | 0.18^o^  (0.05-0.47) | 5.09^o^  (2.20-5.76) |
| **Integrin β7^+^CD39^+^**  **median (IQR)** | 0.39^a,b,c,d,k^  (0.08-0.71) | 11.15^k^  (7.48-18.50) | 4.42^a,l^  (2.56-7.01) | 14.15^l^  (8.78-18.95) | 2.27^b,h,m^  (1.45-4.76) | 9.52^m^  (5.86-16.65) | 6.04^c,h,n^  (4.14-7.33) | 10.74^n^  (9.18-13.15) | 5.12^d,o^  (1.42-9.94) | 10.25^o^  (8.48-14.65) |
| **Integrin β7^+^LAP(TGF-β1)^+^ median (IQR)** | 0.82^a,c,d,k^  (0.43-2.13) | 32.90^b,k^  (26.85-38.93) | 2.48^a,f,l^  (1.15-4.23) | 31.54^e,l^  (22.50-39.43) | 2.01^h,m^  (1.28-2.72) | 23.66^b,e,h,m^  (15.56-26.30) | 5.02^c,f,h,n^  (4.04-9.35) | 32.31^h,n^  (26.26-36.20) | 4.45^d,o^  (1.21-8.03) | 26.99^o^  (20.15-36.53) |

**Supplementary Table 2.** Comparison of the expression of analysed sub-population among total CD8 T-cells *versus* FoxP3^+^ CD8 T-cells in cross-sectional study groups.

Results are shown as median and interquartile range (IQR).

Significant differences (p < 0.05) following Mann–Whitney for the comparison between total CD8 T-cells or FoxP3^+^ CD8 T-cells within different study groups are mentioned as follow: **a**: Non-infected vs Acute, **b**: Non-infected vs Chronic (ART-), **c**: Non infected vs Chronic (ART+), **d**: Non-infected vs EC, **e**: Acute vs Chronic (ART-), **f**: Acute vs Chronic (ART+), **g**: Acute vs EC, **h**: Chronic (ART-) vs Chronic (ART+), **i**: Chronic (ART-) vs EC, **j**: Chronic (ART+) vs EC.

Significant differences (p < 0.05) following Wilcoxon signed-rank test for the comparation between total CD8 T-cells *versus* FoxP3^+^ CD8 T-cells among the same study group are mentioned as follow: **k**: Non infected (within total CD8^+^ vs within FoxP3^+^ CD8^+^), **l**: Acute (ART-) (within total CD8^+^ vs within FoxP3^+^ CD8^+^), **m**: Chronic (ART-) (within total CD8^+^ vs within FoxP3^+^ CD8^+^), **n**: Chronic (ART+) (within total CD8^+^ vs within FoxP3^+^ CD8^+^), **o**: EC (within total CD8^+^ vs within FoxP3^+^ CD8^+^)
